# Optimizing metabolic stability of phosphodiesterase 5 inhibitors: Discovery of a potent N‐(pyridin‐3‐ylmethyl)quinoline derivative targeting synaptic plasticity

**DOI:** 10.1002/alz.083623

**Published:** 2025-01-09

**Authors:** ELISA ZUCCARELLO, Hong Zhang, Erica Acquarone, Shi‐Xian Deng, Donald W. Landry, Ottavio Arancio, Jole Fiorito

**Affiliations:** ^1^ Columbia University, New York, NY USA; ^2^ Department of Medicine ‐ Columbia University, New York, NY USA

## Abstract

**Background:**

There are no cures for Alzheimer’s disease (AD), a progressive neurodegenerative disorder characterized by elevation of beta‐amyloid and tau proteins besides neuronal death and causing cognitive impairment. Phosphodiesterase 5 (PDE5) is a cyclic guanosine monophosphate‐degrading enzyme involved in numerous biological pathways including those relevant to memory formation. PDE5 inhibition offers the potential to attenuate AD progression by acting at the downstream level of beta‐amyloid and tau elevation. None of the existing PDE5 inhibitors has been developed to be used in CNS diseases or possesses the selectivity required for chronic administration to an elderly population with comorbid conditions such as AD patients. We now propose to find PDE5 inhibitors (PDE5Is) that are tailored to the use in AD patients

**Method:**

Our strategy used a combination of drug discovery, biochemical and electrophysiological techniques. We identified the quinoline scaffold of our lead compound **7a**, 4‐((3‐chloro‐4‐methoxybenzyl)amino)‐8‐cyclopropyl‐3‐(hydroxymethyl)quinoline‐6‐carbonitrile, as a starting scaffold for a structure activity relationship study aimed at identifying the suitable isosteric substitutions, with the purpose of obtaining new PDE5Is that are more stable than compound 7a, while maintaining potency and selectivity towards other PDE isoforms. The most potent PDE5Is, showing an IC_50_ in the nano‐molar range, were advanced toward an *in vitro* metabolic stability assay, and compounds with the highest potency and microsomal stability were tested for phase I and II metabolic identification. Out of these small molecules, the most potent and stable quinoline derivate, compound 4b, was tested in electrophysiological experiments assessing long‐term potentiation (LTP), a type of synaptic plasticity thought to underlie memory formation.

**Result:**

The characterization of compound **7a** properties revealed high PDE5 potency (IC_50_ = 0.27 nM) and great selectivity, but low *in vitro* human microsomal metabolic stability (half‐life = 20.5min). The second‐generation PDE5Is, compound **4b (**8‐cyclopropyl‐3‐(hydroxymethyl)‐4‐(((6‐methoxypyridin‐3‐yl)methyl)amino)quinoline‐6‐carbonitrile), (IC_50_ = 20 nM) had improved *in vitro* microsomal stability (t_1/2_ = 44.6 min), and an outstanding phase I and II metabolic profile. Both compounds ameliorated the deficit in LTP, a cellular surrogate of memory, after exposure of hippocampal slices to tau oligomers.

**Conclusion:**

These data suggest that compounds **7a** and **4b** are important leads in the pharmacologic restoration of cell‐to‐cell communication and memory in AD.

